# Cam-Based Simple Design of Constant-Force Suspension Backpack to Isolate Dynamic Load

**DOI:** 10.3390/biomimetics10090607

**Published:** 2025-09-10

**Authors:** Haotian Ju, Zihang Guan, Junchen Liu, Yao Huang, Kerui Sun, Lele Li, Weimao Wang, Tianjiao Zheng, Quan Xiong, Jie Zhao, Yanhe Zhu

**Affiliations:** 1 State Key Laboratory of Robotics and Systems, Harbin Institute of Technology, Harbin 150001, China; juhaotian_hit@foxmail.com (H.J.); guanzihang_hit@foxmail.com (Z.G.); liujc_hit@163.com (J.L.); huangyao_hit@foxmail.com (Y.H.); 21b908043@stu.hit.edu.cn (K.S.); 23B908056@stu.hit.edu.cn (L.L.); wangweimao_hit@foxmail.com (W.W.); zhengtj@hit.edu.cn (T.Z.); jzhao@hit.edu.cn (J.Z.); 2 Department of Electromechanical Engineering and Centre for Artificial Intelligence and Robotics, University of Macau, Macau SAR 999078, China; quan.xiong@u.nus.edu

**Keywords:** suspended backpack, constant-force mechanism, load carriage, metabolic cost

## Abstract

Prolonged load carriage with ordinary backpacks (OBs) can cause muscle fatigue and skeletal injuries. Research indicates that suspended backpacks can effectively reduce energy expenditure; however, existing elastic rope-based suspension backpacks struggle to adapt to different speeds, while active suspension backpacks gain significant additional weight due to the incorporated motors and batteries. This paper presents a novel cam-based constant-force suspension backpack (CCSB). The CCSB employs a cam–spring mechanism with near-zero suspension stiffness to minimize the inertial forces generated by load oscillations. A test platform was constructed to evaluate the constant-force performance of the mechanism, showing a maximum error of less than 1.96%. Load-carrying experiments were conducted at different walking speeds. Laboratory test results show that, compared with OBs, the CCSB reduces peak accelerative vertical force by an average of 84.47% and reduces human metabolic costs by 10.58%. Outdoor tests show that the CCSB can reduce transportation consumption by 8.26%. The CCSB’s compact structure makes it more suitable for commercialization and demonstrates significant potential for practical applications.

## 1. Introduction

With the continuous advancement of technology, wearable smart devices have experienced rapid development and widespread adoption [1,2,3]. Carrying loads with backpacks is an essential activity in daily life, particularly when hands need to be free for other tasks while transporting heavy items [4]. However, prolonged load carriage can lead to shoulder strain, muscle fatigue, and skeletal injuries [5]. Traditional backpack designs have primarily focused on ergonomics and balanced pressure distribution to minimize load pressure [6]. Research has shown that during load carriage, the human body’s center of mass oscillates vertically, causing the load to move up and down as well [7]. The human body must bear both static and dynamic loads, resulting in increased metabolic energy expenditure [8]. In other words, under the same body mass condition, the energy expenditure rate increases proportionally with both increasing walking speed and increasing load weight [9]. Inspired by the bamboo pole-carrying method used in Southeast Asia, Rome et al. [10,11] developed a novel load-carrying backpack. The backpack employs elastic cords to suspend the load, which can reduce human energy consumption by 6.2% when the load is 15 kg. A properly designed suspension system with appropriate natural frequency can reduce walking energy expenditure [10,12,13,14]. Following this, multiple researchers have designed similar suspended backpacks [5,13,15,16,17].

To explore the principles of suspended backpacks, Hoover et al. [18] evaluated backpack performance using mass–spring–damper modeling methods. Their findings indicated that the suspension stiffness should be less than half the resonance stiffness to minimize dynamic loads at given walking speeds. Li et al. [19] proposed a model for quantitatively assessing the effects of backpack stiffness and damping on human energetics. The analysis indicates that energy consumption increases near the resonance frequency, and it is even higher at greater walking speeds or with heavier loads. Hou et al. [20] proposed a biomechanical model to analyze the interaction between the human body and energy harvesting backpacks. This model accurately predicted the relationship between human walking parameters and backpack performance. Ackerman et al. [21] analyzed energy relationships across different walking speeds and loads with various backpack types, demonstrating that low suspension stiffness and high mass loads are beneficial for reducing peak accelerative vertical forces.

To accommodate varying human speeds, some active suspension backpacks have been proposed [22,23,24,25]. Yang et al. [22] developed a backpack with motor-driven limit stops to adjust spring stiffness within a specified range. Leng et al. [15] implemented a motor-driven nut mechanism to compress circular washers, thereby modulating spring stiffness and achieving adjustable suspension stiffness. Lin et al. [23,26] engineered a suspended backpack with motor-driven active suspension stiffness adjustment. He et al. [5] designed an active suspension backpack with motor compensation for accelerative vertical forces, significantly reducing load acceleration. However, these backpacks are relatively heavy, increasing additional metabolic energy expenditure. Moreover, the requirement for lithium batteries to power the motors substantially diminishes their practical utility.

To address these limitations, this paper explores bioinspired approaches to suspension system design. It is noteworthy that birds exhibit a head stabilization mechanism that is very similar to a suspended backpack, where the bird is able to keep its head stable relative to its spatial position during locomotion, even when the body displacement and acceleration are highly variable [27]. It has been shown that the specialized musculature of the bird’s neck and the neural control system form an efficient biological vibration isolation system. Among them, the bird neck muscle system utilizes tendon elastic energy storage and the nonlinear properties of the muscle length–tension relationship to achieve energy-efficient vibration isolation. Therefore, designing a flexible suspension similar to bird neck muscles will realize more efficient dynamic load isolation. We propose maximizing the reduction in suspended backpack stiffness to develop a suspension system adaptable to various human movement frequencies. Mechanical factors such as weight and frame thickness must be considered, and lightweight and compact design strategies must be implemented to enhance the effectiveness of load-carrying assistance. A simple and reliable backpack structure will provide an effective guarantee for its commercialization.

The structure of this paper is as follows: Section 2 establishes the dynamic model of the backpack–human system and explains the energy advantages of low-stiffness suspension backpacks. Section 3 presents the design of a constant-force suspension backpack based on a cam–spring mechanism. Section 4 validates the effectiveness through constant-force mechanism experiments, accelerative vertical force tests, load displacement tests, uphill walking tests, and human metabolic energy experiments. Section 5 concludes this article and looks into future work.

## 2. Dynamical Modeling and Analysis

**Figure 1:**
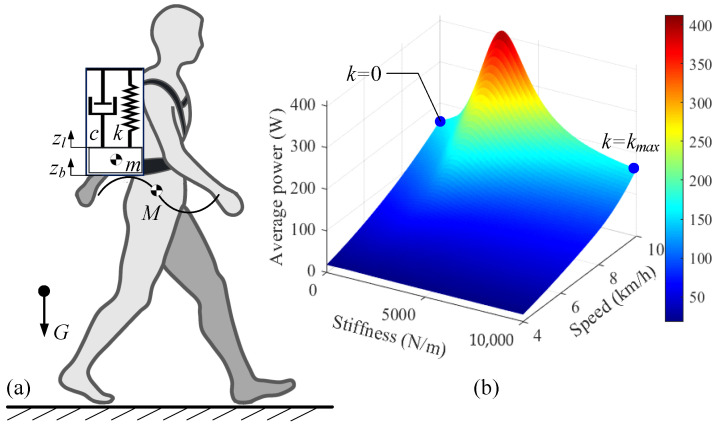
(**a**) Simplified suspended backpack model. (**b**) Power consumption under various speeds and spring stiffness conditions.

Since the impact of load on the human body primarily occurs in the vertical direction [28], the suspended backpack can be represented by a simple spring–mass–damper model, as shown in Figure 1a. The human body’s center of mass exhibits vertical oscillations during gait cycles, primarily resulting from leg extension, which can be approximated as sinusoidal motion [29]:

(1) $$z_{b}\left(t\right)=Z_{b}\sin\left(\omega_{h}t\right)$$

where $Z_{b}$ represents the amplitude of motion, which is related to leg length, *L*, speed, *v*, and frequency, $\omega_{h}$ [30,31], and can be expressed as

(2) $$Z_{b}=\frac{L}{2}\left(1-\sqrt{1-{\left(\frac{0.963\pi v}{L\omega_{h}}\right)}^{2}}\right)-0.0157L$$

The walking frequency ω is related to the human’s movement speed $v_{h}$ and height $s_{h}$ and can be expressed as [21]

(3) $$\omega_{h}=\frac{4\pi \times 64.8{\left(\frac{v_{h}}{s_{h}}\right)}^{0.57}}{60}$$

According to Newton’s Second Law, the dynamic equations for the suspended backpack and human body can be expressed as

(4) $$\left\{\begin{array}{c} m{\ddot{z}}_{l}=k\left(z_{b}-z_{l}\right)+c\left({\dot{z}}_{b}-{\dot{z}}_{l}\right)\\ M{\ddot{z}}_{b}=F_{leg}-k\left(z_{b}-z_{l}\right)-c\left({\dot{z}}_{b}-{\dot{z}}_{l}\right)-mg-Mg \end{array}\right.$$

where *m* represents the load mass, *k* represents the system stiffness, *c* is the damping coefficient, and $F_{leg}$ is the ground reaction force. $z_{b}$ and $z_{l}$ represent the displacement of the human body and load, respectively. The load motion can be characterized by $z_{l}=Z_{l}\sin\left(\omega_{h}t-\beta \right)$. $Z_{l}$ denotes the load motion amplitude, and β represents the phase shift. The load amplitude $Z_{l}$ can be formulated as

(5) $$Z_{l}=Z_{b}\sqrt{\frac{k^{2}+c^{2}{\omega_{h}}^{2}}{{\left(m{\omega_{h}}^{2}-k\right)}^{2}+c^{2}{\omega_{h}}^{2}}}$$

The phase shift β can be expressed as

(6) $$\beta =\arctan(\frac{mc{\omega_{h}}^{3}}{k^{2}+c^{2}{\omega_{h}}^{2}-mk{\omega_{h}}^{2}})$$

The driving forces generated by the legs result in vertical motion of the body’s center of mass. Since the body motion is equated to a sinusoidal motion, only the load force on the legs is considered. It can be expressed as

(7) $$F_{l}=mg+m{\ddot{z}}_{l}$$

The instantaneous power output of the legs can be approximated as the product of the ground reaction force exerted by the legs on the body and the velocity of movement [32]. The power consumption of the human body when carrying a load can be expressed as

(8) $$P_{l}=F_{l}Z_{b}\omega_{h}\cos\left(\omega_{h}t\right)$$

The mechanical work *W* performed over one gait cycle, *T*, can be calculated as the integral of power, where positive work is generated when the leg force aligns with the center-of-mass velocity and negative work when they are antiparallel [19,33,34]. We do not consider the effects of thermoregulation and cardiovascular components on energy. *W* can be formulated as

(9) $$W=\int_{0}^{T}P_{l}/\eta dt=\int_{0}^{\frac{2\pi }{\omega_{h}}}P_{l}/\eta dt$$

where η represents the efficiency of mechanical work performed by muscles, with an efficiency of 25% for positive work and −120% for negative work [8,19]. η can be expressed as

(10) $$\eta =\left\{\begin{array}{c} 25\%\;(P_{l}\geq 0)\\ -120\%\;(P_{l}<0) \end{array}\right.$$

The average mechanical power generated during a human gait cycle can be expressed as

(11) $${\tilde{P}}_{l}=\frac{W}{T}$$

The parameters of the human body and the backpack are presented in Table 1. The relationship between walking speed, spring stiffness, and power consumption under constant mass was simulated, as shown in Figure 1b. The blue point represents a typical point of stiffness and power variation at 10 km/h. The variation in energy consumption at different speeds is notably significant, with energy consumption continuously increasing as speed increases. Under constant load weight, energy consumption first increases and then decreases as spring stiffness increases. Resonance occurs when the body’s motion frequency is the same as the load’s intrinsic frequency. The load will generate more significant displacement and acceleration, increasing the body’s energy consumption. Although increasing backpack stiffness can theoretically reduce energy consumption, it compromises wearing comfort. Therefore, maximizing the reduction of backpack stiffness emerges as a more optimal solution.

## 3. Structural Design

**Figure 2:**
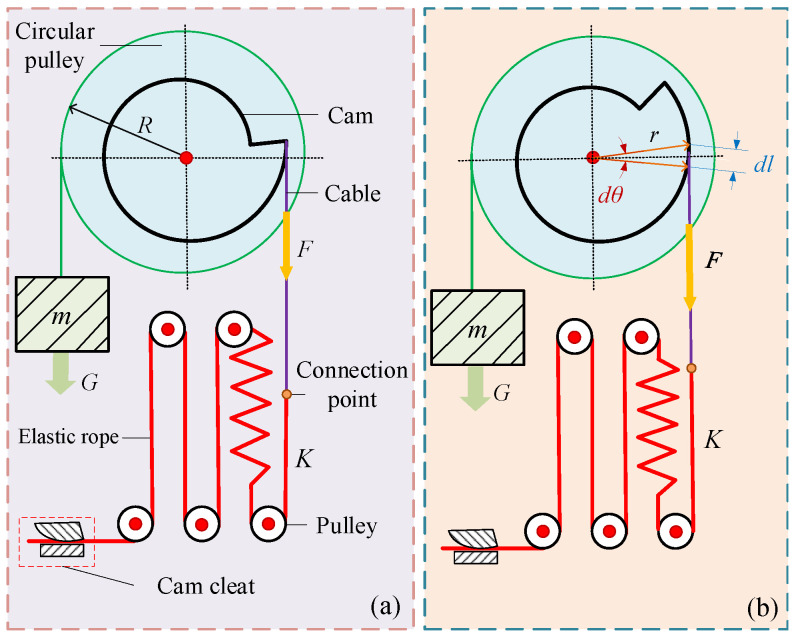
Schematic diagram of cam–spring constant-force mechanism. (**a**) Initial moment equilibrium state. (**b**) Dynamic moment equilibrium during rotation.

A cam–spring mechanism based on the gravity balancing principle [35] was designed to achieve near-zero suspension stiffness of the system, as shown in Figure 2. The circular pulley is connected to the load through a cable. The circular pulley and cam are coaxially connected. The elastic rope is connected to the cam via a cable, which wraps around the cam. The mechanism achieves a constant pulling force on the load through a customized cam profile design. When the net external force on the load is zero, the load will remain stationary relative to the ground, achieving complete cancellation of the accelerative vertical force.

When the weight of the load equals the pulling force of the cam–spring mechanism, the resultant external force on the load is zero, resulting in equivalent zero suspension stiffness. Endo et al. [36] proposed a numerical–iterative method for the rapid determination of such cam profile curves. However, this method cannot reliably guarantee shape convergence and is highly sensitive to the selection of design parameters, requiring extensive trial and error during the design process. Building on the analytical formulation of the cam profile, Ludovico et al. [37] introduced a convex-optimization approach capable of producing feasible solutions for arbitrary target torque profiles. Nevertheless, this approach incurs a moderate loss of accuracy compared to closed-form analytical solutions, and its computational complexity grows markedly with the increase in polynomial fitting order. To overcome these limitations, we propose a simplified analytical method for approximating cam profiles, which employs fewer adjustable design parameters and entails significantly lower computational complexity.

Based on force equilibrium, the moment balance equation of the system can be expressed as

(12) $$mgR=Fr\left(\theta \right)$$

where *R* is the radius of the circular pulley, θ is the rotation angle of the load, *F* is the spring tension force, and r(θ) is the radius of the cam at the corresponding rotation angle. Differentiating both sides of the equation with respect to θ yields

(13) $$0=F\frac{dr}{d\theta }+r\frac{dF}{d\theta }$$

The tension force exerted by the spring can be formulated as $F=F_{0}-Kl$, where $F_{0}$ is the initial spring tension force, *K* is the spring stiffness, and *l* is the spring extension. It follows from the differential relation:

(14) $$\left\{\begin{array}{c} dF=-Kdl\\ dl=rd\theta \end{array}\right.$$

Substituting Equation (14) into Equation (13) yields

(15) $$\frac{mgR}{Kr^{3}}dr=d\theta$$

The boundary condition is $r\left(0\right)=r_{0}$. *r* can be expressed as

(16) $$r={\left(\frac{1}{r_{0}^{2}}-\frac{2K}{mgR}\theta \right)}^{-\frac{1}{2}}\left(\theta \leq \frac{mgR}{2Kr_{0}^{2}}\right)$$

**Figure 3:**
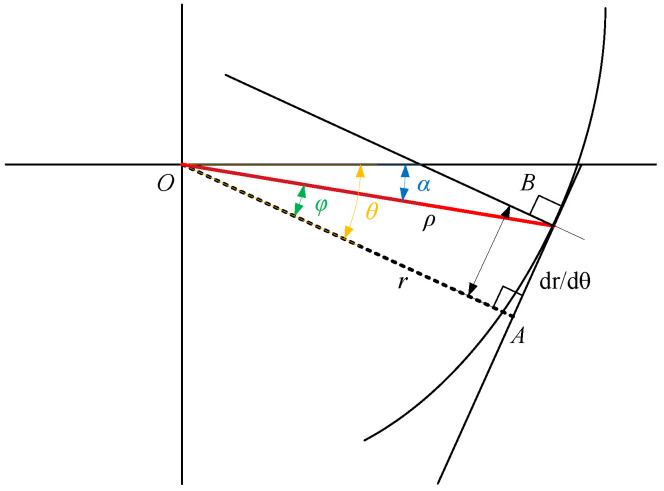
Error analysis of cam design.

Since the cam contour line is not circular, the position of the normal line on the actual contour has an offset distance with respect to the force arm, resulting in the force arm and the actual cam radius vector not coinciding, as shown in Figure 3. *B* is the actual point of separation of the cable from the cam, i.e., the point at which the cable is tangent to the cam contour. The radius vector of the cam, |OB|, is denoted as ρ, |OA| is the actual force arm of the cable *r*, and the slope of the line where the cable is located is

(17) $$\frac{dy}{dx}=\frac{d\left(\rho \sin\alpha \right)}{d\left(\rho \cos\alpha \right)}=\frac{\left(\rho \cos\alpha +\frac{d\rho }{d\alpha }\sin\alpha \right)d\alpha }{\left(-\rho \sin\alpha +\frac{d\rho }{d\alpha }\cos\alpha \right)d\alpha }=\frac{\rho \cos\alpha +\frac{d\rho }{d\alpha }\sin\alpha }{-\rho \sin\alpha +\frac{d\rho }{d\alpha }\cos\alpha }$$

The line AB can be expressed as

(18) $$Px+Qy+\rho^{2}=0$$

where $P=-\left(\rho \cos\alpha +\frac{d\rho }{d\alpha }\sin\alpha \right)$, $Q=-\rho \sin\alpha +\frac{d\rho }{d\alpha }\cos\alpha$.

|OA| can be expressed as

(19) $$\left|OA\right|=\frac{\left|\rho^{2}\right|}{\sqrt{P^{2}+Q^{2}}}=\frac{\rho^{2}}{\sqrt{\rho^{2}+{\left(\frac{d\rho }{d\alpha }\right)}^{2}}}=\rho \frac{1}{\sqrt{1+{\left(\frac{\rho^{\prime }}{\rho }\right)}^{2}}}$$

In a right triangle, ABO, by the Pythagoras theorem,

(20) $$\left|AB\right|=\sqrt{{\left|OB\right|}^{2}-{\left|OA\right|}^{2}}=\frac{\rho \frac{d\rho }{d\alpha }}{\sqrt{\rho^{2}+{\left(\frac{d\rho }{d\alpha }\right)}^{2}}}=\rho^{\prime }\frac{1}{\sqrt{1+{\left(\frac{\rho^{\prime }}{\rho }\right)}^{2}}}$$

|OB| can be expressed as

(21) $$\left|OB\right|=\sqrt{\rho^{2}+\rho^{\prime 2}}\frac{1}{\sqrt{1+{\left(\frac{\rho^{\prime }}{\rho }\right)}^{2}}}$$

When $\left|\frac{\rho^{\prime }}{\rho }\right|\ll 1$, $\frac{1}{\sqrt{1+{\left(\rho^{\prime }/\rho \right)}^{2}}}\approx 1$. When |OA| is the force arm r(θ), the offset distance |AB| can be expressed as

(22) $$\left|AB\right|=\frac{dr}{d\theta }=\frac{K}{mgR}{\left(\frac{1}{r_{0}^{2}}-\frac{2K}{mgR}\theta \right)}^{-\frac{3}{2}}$$

The radius vector |OB| of the actual cam can be expressed as

(23) $$r_{a}={\left(\frac{1}{r_{0}^{2}}-\frac{2K}{mgR}\left(\theta +\varphi \right)\right)}^{-\frac{3}{2}}\sqrt{{\left(\frac{1}{r_{0}^{2}}-\frac{2K}{mgR}\left(\theta +\varphi \right)\right)}^{2}+{\left(\frac{K}{mgR}\right)}^{2}}$$

The angle ϕ through which the cam’s radius vector, |OB|, rotates relatively to r(θ) is

(24) $$\varphi \left(\theta \right)=\arctan\left(\frac{dr}{rd\theta }\right)=\arctan\left[\frac{K}{mgR}{\left(\frac{1}{r_{0}^{2}}-\frac{2K}{mgR}\theta \right)}^{-1}\right]$$

In order to ensure the validity of the above calculations, the constraint $\left|\frac{r^{\prime }}{r}\right|\leq \varepsilon <1$, solving for the range of angles for which the approximation is valid, is $\theta \leq \frac{mgR}{2Kr_{0}^{2}}-\frac{1}{2\varepsilon }<\frac{mgR}{2Kr_{0}^{2}}$, which indicates that the cam profile obtained by the above approximation is exists globally.

Consider the initial condition $r\left(0\right)=r_{0}$. To simplify the computation of the initial condition of the mechanism, so that θ=0 is within the above valid range, we have $\frac{mgR}{2Kr_{0}^{2}}-\frac{1}{2\varepsilon }\geq 0$, which solves for $\varepsilon \geq \frac{Kr_{0}^{2}}{mgR}$.

The pre-stretch of the spring $l_{0}$ is

(25) $$l_{0}=\frac{mgR}{Kr_{0}}$$

**Figure 4:**
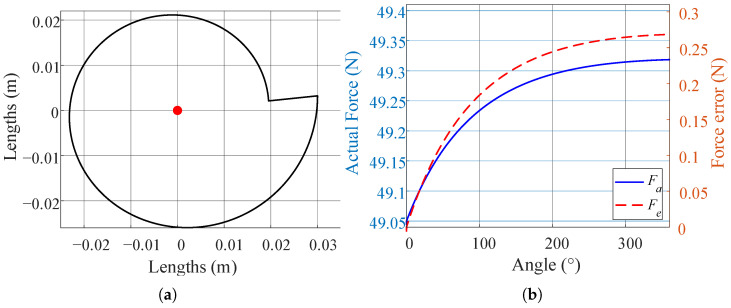
(**a**) Contour line of the cam. The red point indicates the center of rotation of the cam. (**b**) Relationship between cam angle change and balanced load weight. $F_{a}$ is the actual force and $F_{e}$ is the error between the actual and target values.

The target load of the designed suspended backpack is 10 kg. Two sets of symmetric constant-force mechanisms are used to balance the load, and each constant-force mechanism balances a weight of 5 kg. The gravitational acceleration is taken as 9.81 m/s^2^. The radius of the circular pulley is 0.04 m, and the elastic rope stiffness is about 235 N/m. The initial condition is set as $r_{0}$ = 0.03 m; then the minimum value of ϵ is about 0.108, and it can be assumed that the approximation is effective under the condition. The initial tensile force is about 65.4 N, the pre-stretching length is 278.3 mm, and the contour curve of the cam is plotted in the range of [0, 2π], and the calculated contour curve of the cam is shown in Figure 4a. Considering that the cam will interfere with the cable when its rotation reaches a certain angle, the final working range of the cam is [0, 1.5π], and the total distance of the load’s up-and-down displacement is 180 mm.

The relationship between the angle change and the balanced load weight is shown in Figure 4b; with the increase in the angle, the error of the balanced load weight gradually rises but tends to level off, and the maximum error is only 0.27 N, which indicates that the cam design method proposed in this paper has good accuracy.

**Figure 5:**
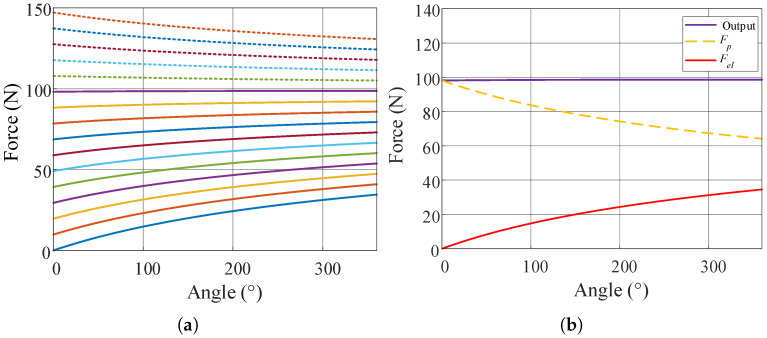
(**a**) The relationship between the output force *F* and the cam angle, for different initial forces, $F_{0}$ (dashed lines indicate loads exceeding 10 kg, and solid lines indicate loads less than 10 kg). (**b**) Under 10 kg load equilibrium conditions, the variation in $F_{p}$ and $F_{el}$ with angle.

In order to study the usable load range of the suspended backpack, the initial spring tension $F_{0}$ is adjusted for simulation, and the balancing effect under different initial tensions is shown in Figure 5a. In the process of cam rotation, the force *F* output from the mechanism can be split into two parts; one is determined by the preload force $F_{p}$, and the other is determined by the elastic deformation force $F_{el}$ caused by the winding of the cable on the cam. The output force *F* can be expressed as

(26) $$F=F_{p}+F_{el}=\frac{1}{R}\cdot F_{0}\cdot r\left(\theta \right)+\frac{1}{R}\cdot Kl\left(\theta \right)\cdot r\left(\theta \right)$$

The system stiffness decreases with increasing load weight. In the 10 kg load range, it shows positive stiffness characteristics. After exceeding 10 kg, the system stiffness becomes negative. The change in system stiffness is mainly due to the opposite trends of $F_{p}$ and $F_{el}$. The contribution of these two components of force in the output changes as the initial tension changes as a percentage of the tension on the cable, as shown in Figure 5b. When the equilibrium load is less than 10 kg, the corresponding $F_{0}$ is smaller and the spring tension accounts for a larger share, thus showing a positive stiffness characteristic; when the load is larger than 10 kg, the initial tension accounts for a larger share and the change is more obvious, showing a negative stiffness characteristic. When the backpack suspension has positive stiffness characteristics, although it can still reduce the inertial force of the load, the load reduction effect is lower compared to that with a 10 kg load. When the backpack suspension has negative stiffness characteristics, the load will gradually move downward and eventually collide with the slide limits. This situation cannot reduce the inertial force and may cause discomfort for the user. Therefore, the working range of the suspended backpack designed in this paper is no more than 10 kg.

**Figure 6:**
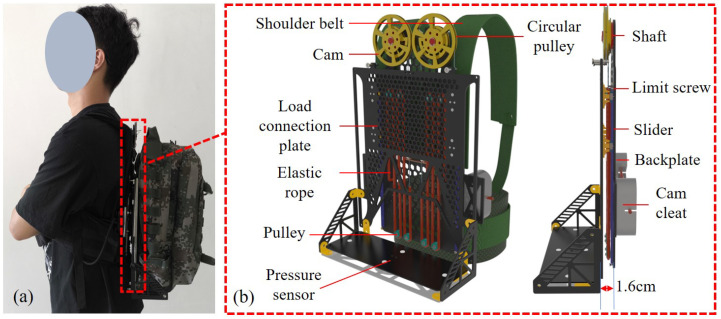
Designed suspended backpack. (**a**) Subject wearing the designed suspended backpack. (**b**) Side view of the designed suspended backpack. The distance between the connection plate and the backplate is only 1.6 cm, which greatly reduces the overturning moment of the load on people.

The structure of the suspended backpack is shown in Figure 6. The overall structure is made of carbon fiber material, with key components made of aluminum alloy, and the total weight does not exceed 1.2 kg. Two sets of symmetrical constant-force mechanisms are arranged on both sides of the backplate. The load connection plate is restricted to vertical movement along the guide rails, and a limiting structure is installed at the end of the guide rails to prevent the load from slipping out of the rails. The system can withstand a vertical load displacement of 180 mm. According to the model in Section 2, when a person’s speed reaches 15 km/h, the relative displacement between the backpack and the person exceeds the rail limits; therefore, the maximum speed limit for using this backpack is 15 km/h. One end of the elastic rope is fixed to the cam cleat, while the other end passes through multiple pulleys and connects to the cable. The elastic rope and cable are secured using wire clips. The cable is secured to the cam. The cable can move within the cam’s groove. A cable connects the circular pulley to the load connection plate. The circular pulley and cam are coaxially fixed. The canvas backpack is secured to the load connection plate via straps. The load connection plate is equipped with a pressure sensor to detect the load weight. A 10 kg load is placed inside the backpack.

## 4. Experimental Performance Evaluation

### 4.1. Constant-Force Mechanism Experiments

**Figure 7:**
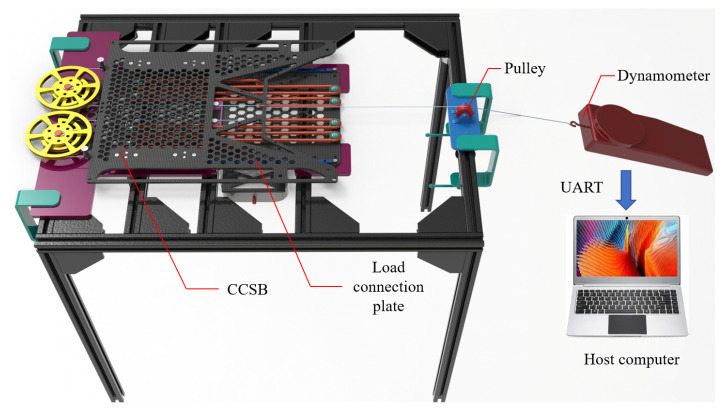
Testing platform for the constant-force mechanism.

A tensile test was conducted on the mechanism to verify the accuracy of the constant-force mechanism design. The test platform is shown in Figure 7. The suspended backpack was securely mounted on the test bench using screws. A wire rope connected to a dynamometer was attached to the load connection plate. A fixed pulley was utilized to prevent force deviation during the pulling process. The load connection plate was pulled from 0 mm to 180 mm using a dynamometer at a velocity of 0.2 m/s, followed by releasing the wire rope at the same velocity. Force sensor data was transmitted to the host computer via a serial port for acquisition and storage. The experiment was repeated three times, and the average values were calculated.

**Figure 8:**
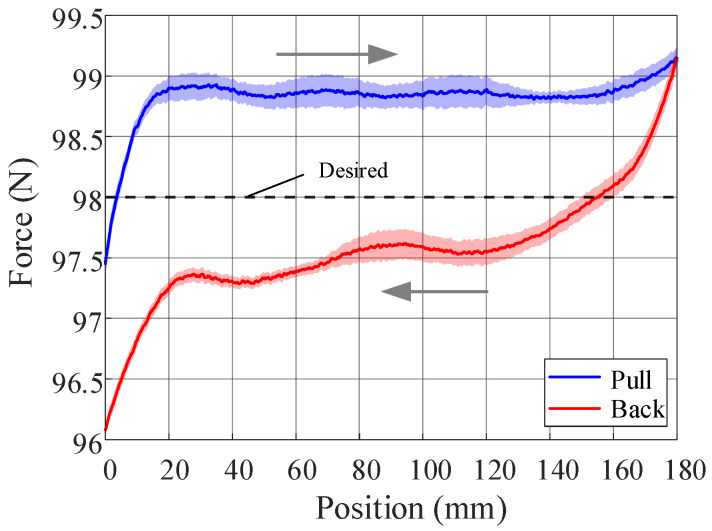
The tension test results of the cam–spring constant-force mechanism. The arrows indicate the movement direction of the load connection plate.

Figure 8 presents the experimental results. During the pulling phase, the tension stabilized at 98.85 N in the constant-force stage, with a relative error of 0.87% and a maximum force error of 1.17%. In the release phase, the tension stabilized at 97.54 N, with a relative error of 0.47% and a maximum force error of 1.96%. These results indicate that the designed constant-force mechanism has a good constant-force effect. The discrepancies are mainly due to assembly errors and friction between the guide rail and slider.

### 4.2. Human Experiment

#### 4.2.1. Experimental Methods

**Figure 9:**
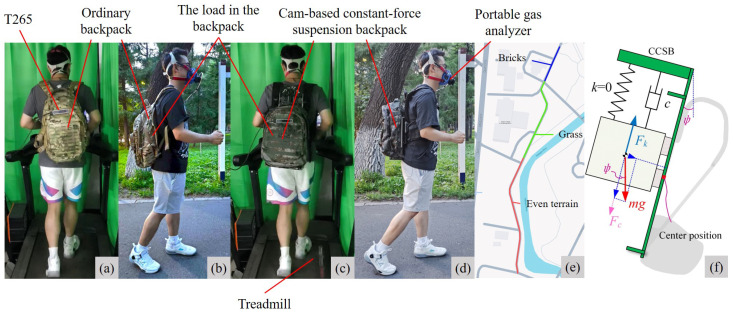
Experimental procedure. (**a**,**c**) show participants wearing ordinary backpacks and suspended backpacks, respectively, while walking on a treadmill with a load. (**b**,**d**) show participants wearing ordinary backpacks and suspended backpacks, respectively, while walking outdoors with a load. The location of the load under different conditions was the same, in the middle of the back. (**e**) Map of the walking route used in the outdoor experiment. (**f**) Force distribution on the load when the human body leans forward.

Eight healthy adult males without gait abnormalities participated in this experiment to evaluate the load-reducing effectiveness of the designed suspended backpack. Participant baseline data were as follows: mean age was 25 ± 3 years, mean body weight was 65 ± 4 kg, and mean height was 178 ± 5 cm. As shown in Figure 9a–d, laboratory and outdoor load-bearing experiments were conducted. The laboratory experiments evaluated the performance of the backpack during steady-state walking, while the outdoor experiments assessed its feasibility in real-world applications. The laboratory experiments included accelerative vertical force experiments, displacement experiments, uphill walking experiments, and human energy metabolism experiments. For the outdoor load-bearing experiments, participants completed load-bearing walking on a 420 m sidewalk, choosing their own walking speed. The road surface included grass, cement, and brick, as shown in Figure 9e.

In the laboratory acceleration and displacement experiments, participants carried a 10 kg load using an ordinary backpack (OB) and a cam-based constant-force suspension backpack (CCSB) while walking on a professional treadmill (Zhejiang Umay Technology Co., Ltd., Jinhua, China). The location of the load under different conditions was the same, in the middle of the back. Walking speeds were set at 5 km/h, 7 km/h, and 9 km/h, with each trial lasting 5 min. Data from the final 2 min were selected as steady-state walking-phase samples for analysis. A 20 min rest interval between trials was implemented to mitigate fatigue effects. RealSense T265 tracking cameras (Intel Corporation, Santa Clara, CA, USA) were mounted on both backpack types to record load displacement and acceleration values, transmitting data via serial communication to a host computer at a 200 Hz sampling frequency. Accelerative vertical forces were calculated using F=ma.

To determine how forward trunk inclination affects the load’s displacement, we conducted the uphill walking experiment. With the treadmill set at a 20° incline, the measured forward lean angle of the participant was approximately 20°. The load force distribution when the trunk leans forward is shown in Figure 9f. An external force, $F_{c}=mg\left(1-cos\psi \right)$, acts on the load, since the tensile force $F_{k}$ of the suspension mechanism does not match the component of gravity of the load. ψ indicates the forward lean angle of the CCSB. During the experiment, the initial tension of the elastic rope in the CCSB was adjusted to mgcosψ to set the load in equilibrium. The participant walked on the treadmill with the CCSB unadjusted and adjusted. Walking speeds were set at 5 km/h, with each trial lasting 5 min. Data from the final 2 min were selected as steady-state walking-phase samples for analysis. T265 RealSense tracking cameras were mounted on the backpack to record load displacement values.

During the human energy metabolism experiment in the laboratory, participants carried a 10 kg load and walked on a treadmill at 5 km/h with two backpack types and under three stiffness regimes: CCSB(ON) ≈ near-zero stiffness (automatic load suspension); CCSB(OFF) ≈ maximum stiffness (rigid screw-fixed connection); and an intermediate, finite stiffness represented by the OB. Participants performed 6 min of load-carrying walking in each condition, with the last 3 min analyzed as a steady state. Participants were prohibited from holding treadmill handrails. A portable gas analyzer (Cosmed K5, Rome, Italy) monitored O_2_ and CO_2_ exchange, with the metabolic cost calculated using the Brockway equation (Brockway, 1987) [38]. For outdoor experiments, the energy cost of transportation was obtained by dividing the cumulative metabolic cost by the total walking distance [39,40]. We used paired t-tests and the Holm–Šidák correction [41] to compare the tested conditions with each other, with significance set at 0.05.

All experimental sequences were randomized to prevent muscle memory effects. To ensure the quality of the experiment, all participants were well prepared: they received a 15 min treadmill acclimatization and a 30 min practice session one day prior to formal testing. The study protocol received ethical approval from the Harbin Institute of Technology Medical Ethics Committee, and written informed consent was obtained from all participants.

#### 4.2.2. Experimental Results and Analysis

**Figure 10:**
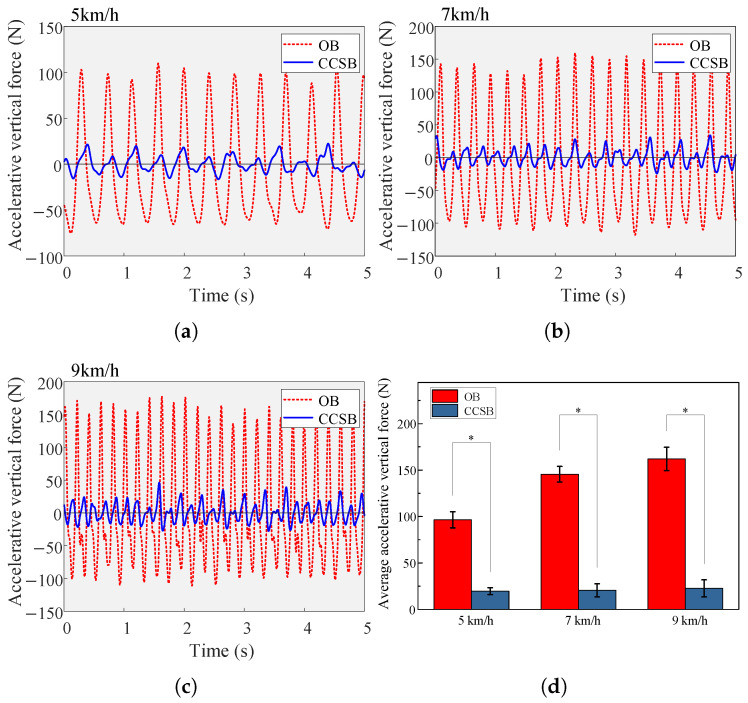
(**a**–**c**) Vertical accelerative forces of a participant wearing two types of backpacks at different speeds. (**d**) Average peak accelerative vertical force at different speeds (mean ± 1 SD). ∗ indicates a significant influence of a backpack condition (*p* < 0.05).

The variation in the accelerative vertical force of the load is shown in Figure 10. Figure 10a–c illustrate the temporal evolution of load accelerative vertical forces post-gait stabilization for a single participant, while Figure 10d depicts the magnitude of the average peak accelerative vertical force of the load.

As locomotion velocity increased, the peak accelerative vertical forces of the load also increased. The CCSB generated accelerative vertical forces of 19.62 ± 3.64 N, 20.49 ± 7.06 N, and 22.63 ± 9.14 N at velocities of 5, 7, and 9 km/h, respectively. Compared to the OB, the CCSB reduced the peak accelerative vertical forces by a maximum of 86.03%, with an average reduction of 84.47%. This demonstrates the effectiveness of the cam-based constant-force system in attenuating accelerative vertical forces across a range of velocities. Additionally, slight residual fluctuations in the inertial forces after the suspension suggested that the elastic rope stiffness’s nonlinearity may have affected the system’s mechanical equilibrium. Frictional interactions between the floating and static components could have contributed to minor fluctuations in the floating assembly.

**Figure 11:**
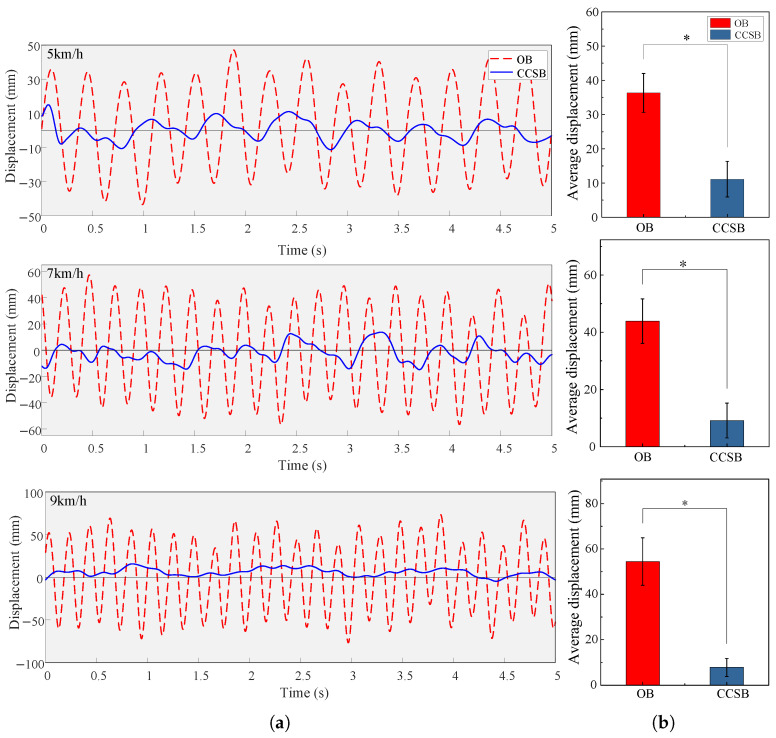
(**a**) Vertical displacement of a participant wearing two types of backpacks at different speeds. (**b**) Average peak vertical displacement of the two backpacks. ∗ indicates a significant influence of a backpack condition (*p* < 0.05).

The displacement results of the load are shown in Figure 11. As the human body’s movement speed increased, the OB’s vertical displacement continued to increase. In contrast, the vertical displacement of the CCSB showed slight variation, with an amplitude of approximately 1 cm. The load excursion of the CCSB at 5, 7, and 9 km/h was 11.11 ± 5.20 mm, 9.20 ± 6.08 mm, and 7.81 ± 3.95 mm, respectively. As movement frequency increased, the load reduction effect improved because higher human motion frequencies increasingly exceeded the natural frequency of the load. Compared with the OB, the CCSB reduced the load excursion by a maximum of 85.67% and an average of 79.13%. This indicates that the constant-force suspension backpack effectively isolated the movement of the human body, keeping the load nearly stationary relative to the ground.

**Figure 12:**
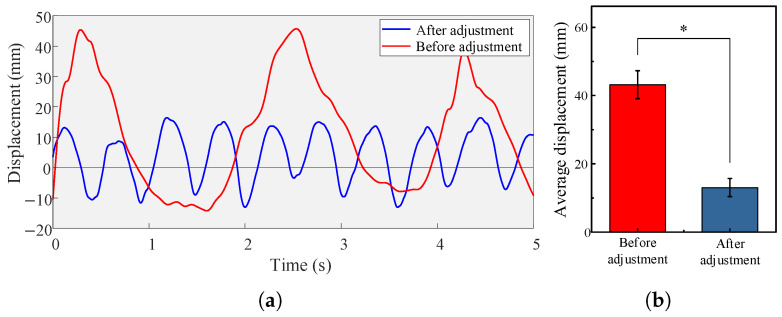
(**a**) Displacement of the load of a participant wearing an unadjusted and adjusted CCSB on the inclined treadmill. (**b**) Average peak vertical displacement of the two backpack models. ∗ indicates a significant influence of a backpack condition (*p* < 0.05).

The experimental results of uphill walking are shown in Figure 12. The load gradually moved upward and struck the rail stop. After impacting the stop, the load decelerated downward and then resumed upward motion to strike the stop again. With the initial tension adjustment, the load no longer collided with the rail stop, indicating that the CCSB exhibited some adaptability to the forward-leaning trunk. However, since the system was no longer in a constant-force equilibrium state, the load displacement became greater compared to the vertical position relative to the back.

**Figure 13:**
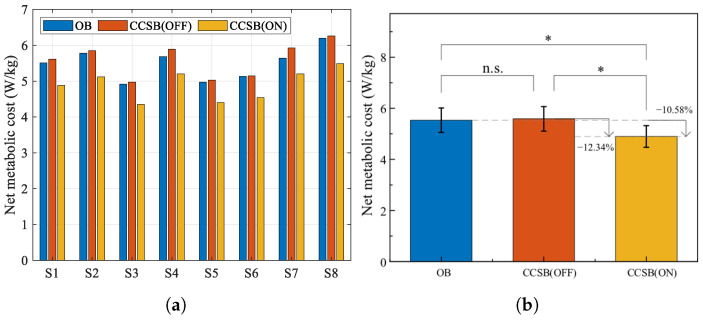
(**a**) The net metabolic rate of each subject. (**b**) The average net metabolic rate of all subjects (mean ± 1 SD; N = 8). The bar represents the mean value and the error bar denotes the standard deviation. ns indicates no significant influence of the backpack condition (*p* > 0.05). ∗ indicates a significant influence of the backpack condition (*p* < 0.05).

Figure 13 shows the results of human energy metabolism in the laboratory. The average net metabolic costs of load carriage were 5.59 ± 0.48 W/kg, 5.48 ± 0.44 W/kg, and 4.90 ± 0.42 W/kg for the CCSB(OFF), OB, and CCSB(ON) conditions, respectively. Comparable metabolic costs were observed between OB and CCSB(OFF), indicating that the weight of the CCSB exerted negligible influence on human energy consumption. Compared with CCSB(OFF), CCSB(ON) demonstrated an 12.34% average reduction in energy expenditure. Compared to OB, CCSB(ON) achieved a 10.58% mean decrease in metabolic cost. Our design’s lower suspension stiffness significantly reduced accelerative vertical forces on the load, consistently with previous findings demonstrating that reducing shoulder impact forces effectively decreases metabolic expenditure [28]. The comparison between the CCSB and some existing suspended backpacks is shown in Table 2. Compared to existing suspended backpacks, the CCSB is the lightest, weighing only 1.2 kg. Huang et al.’s [17] passive suspension backpack (3.2 kg) achieved only an 8.81% metabolic reduction, partly because its greater self-weight increases human energy consumption and partly because its heavier load affects human gait. The CCSB approaches the load-reducing effectiveness of He et al.’s [5] active suspension backpack (10.98%), but without requiring energy consumption from active motor control. The CCSB demonstrates advantages in both lightweight design and energy metabolism.

**Figure 14:**
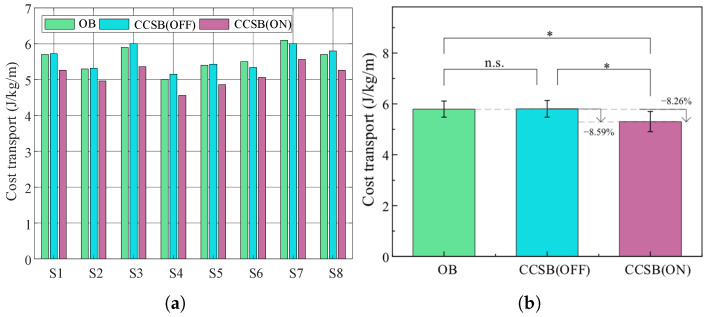
(**a**) Transportation costs for each subject. (**b**) The average of all subjects (mean ± 1 SD; N = 8). The bar represents the mean value and the error bar denotes the standard deviation. ns indicates no significant influence of the backpack condition (*p* > 0.05). ∗ indicates a significant influence of the backpack condition (*p* < 0.05).

For outdoor experiments, the results of energy metabolism are shown in Figure 14. The average energetic costs of transport were 5.59 ± 0.33 J/kg/m, 5.57 ± 0.35 J/kg/m, and 5.11 ± 0.32 J/kg/m for the CCSB(OFF), OB, and CCSB(ON) conditions, respectively. Compared with CCSB(OFF), CCSB(ON) reduced energy consumption by an average of 8.59%. Compared with OB, it reduced energy consumption by an average of 8.26%. The CCSB effectively reduced human energy expenditure during outdoor testing.

During the experiments, we discovered that although CCSB(ON) could decouple the movement of the load from human movement, the state of the load was easily affected by external forces. The stability of the CCSB depends on the balance between the load weight and the suspension’s tensile force. Uneven terrain can affect human movement speed and the angle of trunk tilt. This was verified in the uphill test. When the trunk leans forward, the suspension tension exceeds the load weight, causing the load to move upward until it gradually collides with the stop. Therefore, maintaining an upright trunk enhances the suspension’s vibration isolation effect. When the suspension stiffness approaches zero, the human movement frequency remains higher than the load’s natural frequency, enabling the load to maintain a relatively stationary state and achieve effective vibration isolation. Therefore, speed changes have a limited impact on the stability of the CCSB system. Future work will conduct more detailed experiments on trunk forward lean, further optimize the system’s adaptability in complex terrain conditions, and enhance the CCSB’s stability performance across various challenging environments.

## 5. Conclusions

This study proposes a cam-based constant-force suspension backpack system that utilizes the principle of gravitational balance, with a total mass of 1.2 kg. The system effectively reduces vertical acceleration forces across a range of walking speeds. The CCSB reduced peak vertical acceleration forces by an average of 84.47%, indicating the system’s therapeutic potential in mitigating skeletal muscle injuries caused by high-impact loads. Laboratory comparative analysis revealed that under the same load conditions, the net metabolic rate of the CCSB was reduced by 10.58% compared to that of an ordinary backpack. Outdoor experiments indicated that under the same load conditions, the transportation energy consumption of the CCSB was reduced by 8.26%.

The cam–spring constant-force mechanism is more compact in structure compared to the hinged-lever constant-force mechanism [42], especially when a longer working stroke is required. Therefore, the cam-based suspension backpack is simpler and more compact in structure compared to previous constant-force suspension backpacks [30], making it more suitable for commercialization. By utilizing a specially designed cam profile, the suspension stiffness is nearly zero, significantly reducing impact forces. This results in reduced energy expenditure for the human body during both walking and running. However, the system also has limitations: the constant-force balancing effect is dependent on the cam radius and elastic rope stiffness, making it unable to adapt to loads of different masses perfectly. Although mechanical complexity has been reduced compared to previous designs, it remains higher than that of traditional backpacks, which may increase maintenance requirements and manufacturing costs. Despite these issues, the system’s advantages in reducing inertial forces and metabolic costs indicate significant potential benefits in load-bearing applications. Further research will involve more in-depth dynamic testing of the constant-force mechanism [43], exploring the scalability of this assistive mechanism in other mobility modes, and optimizing the mechanical design to enhance its adaptability under different load conditions.

## Figures and Tables

**Table 1:** The parameters used in simulations.

Parameters	Description	Values
*M*	Body mass (kg)	74
*m*	Load mass (kg)	10
$s_{h}$	Body height (m)	1.78
*L*	Leg length (m)	1
*k*	Stiffness (N/m)	0–10,000
*c*	Damping (Ns/m)	100
*v*	Speed (km/h)	4–10

**Table 2:** Comparison of reduced net metabolic cost between suspended backpacks and ordinary backpacks.

	Our	Huang [17]	Yang [22]	He [5]
Load Mass (kg)	10	15.7	5	19.4
Backpack Mass (kg)	1.2	3.2	2.75	5.3
Passive/Active	Passive	Passive	Semi-active	Active
Reduction (%)	10.58	8.81	4.21	10.98

## Data Availability

The data that support the findings of this study are available from the corresponding author upon reasonable request.

## Abbreviations

The following abbreviations are used in this manuscript:

OB	Ordinary Backpack
CCSB	Cam-based Constant-force Suspension Backpack
